# Supplementary material for: Towards a Treatment for Neuroinflammation in Epilepsy: Interleukin-1 Receptor Antagonist, Anakinra, as a Potential Treatment in Intractable Epilepsy
Source: Int J Mol Sci. 2021 Jun 11;22(12):6282. doi: 10.3390/ijms22126282 (PMC8230637; doi:10.3390/ijms22126282)
Supplement: Supplementary file 1 [file ijms-22-06282-s001.zip › ijms-1250422-supplementary.pdf]

Case	n/Ref.	Onset age (years)	Gender	ASM	Special additions	Sz onset to anakinra (days)	Anakinra dose (mg/kg/day)	Anakinra duration (days)	>50% Sz reduction
1	1/[11]	2y8m	ND	PB, LCM, TPM, PRP, Felbamate, KETA, LEV, PHT, CZP	steroid, KD	6-23, 54-190	5 mg/kg twice daily	190<	yes
2	1/[35]	10y	ND	MDZ, PHT, PRP, TH, PB, LEV, TPM, LCM, CLB, KETA, OXC, PER	IVIg, steroid, KD	540	2.5 mg/kg per day (100 mg) → 2.5 mg/kg twice a day	7 months	yes
3	2/[36]	9y	M	PB, CBD, MDZ, KETA	KD, CMN-DBS	43	titrated up to 5 mg/kg/day	15 months <	yes
4		5y	M	PB, MDZ, TH, KETA, CBD	KD. CMN-DBS	22	titrated up to 10 mg/kg/day	3 months	no
5	1/[37]	21y	F	BZD, LEV, LCM, PENT, PRP, MDZ, KETA, CLB, PER	IVIg, steroid, KD	32	100 mg three times daily	1 year	yes
6	1/[38]	6y	M	LEV, PHT, PB, MDZ, KE , PENT, VPA, TPM, CBD, LZP	IVIg, steroid, Tocilizumab, KD	d6	titrated to 20 mg/kg/day	15 days	no
7	1/[39]	6y	F	LEV, PHT, PB, TPM, LCM, PENT, MDZ, CBD, LID,	IVIg, steroid, KD	24	100 mg twice daily	1 yrs<	yes
8	25/[41]	5.6	M	MDZ, PENT, LID, KE	IVIg, steroid, KD	17	10 mg/kg/day	1 day	yes
9		9	M	MDZ, PENT, LID, CBD, DBS	IVIg, steroid, PE, KD	42	4 mg/kg/day	> 114 days	no
10		5	M	MDZ, PENT, LID, KE, CBD, DBS	IVIg, steroid, PE, KD	21	10 mg/kg/day	> 124 days	no
11		11	M	MDZ, PENT, KE,	IVIg, steroid, PE, KD, HYPO	14	12.2 mg/kg/day	83 days	yes
12		11	F	MDZ, PENT, KE	IVIg, steroid, KD	19	3 mg/kg/day	-	yes
13		5	M	MDZ, PENT, VNS, ISO	IVIg, steroid, PE, KD, HYPO,	18	4.7 mg/kg/day	2 days	-
14		5	F	MDZ, PENT, LID, KE	IVIg, steroid, KD	24	13 mg/kg/day	-	-
15		7	M	MDZ, PENT, KETA	IVIg, steroid, KD	50	6.3 mg/kg/day	330 days	-
16		12	M	MDZ, PENT, PRP, KE, CBD	IVIg, steroid, KD	20	10 mg/kg/day	350 days	-
17		8	M	MDZ, PENT, KE	IVIg, steroid, KD, HYPO	23	2 mg/kg/day	7 days	-
18		11	F	MDZ, PENT, PRP, KE	IVIg, KD	9	8 mg/kg/day	26 days	yes
19		9	F	MDZ, PENT	IVIg, steroid, rituximab	15	3.3 mg/kg/day	14 days	-
20		6	F	MDZ, PENT	KD	d5	10 mg/kg/day	16 days	-

21		9	M	MDZ, PENT	IVIg, steroid, rituximab, KD, HYPO	d20	5 mg/kg/day	120 days	yes
22		6	M	PENT	IVIg, steroid, PE	d32	7 mg/kg/day	252 days	yes
23		15	M	MDZ, PENT	IVIg, steroid, PE	d25	4 mg/kg/day	420 days	-
24		8	M	MDZ, PENT, CBD	IVIg, steroid, HYPO	d6	3.8 mg/kg/day	9 days	no
25		8	M	MDZ	IVIg, steroid, PE, KD	d20	3.2 mg/kg/day	90 days	yes
26		7	M	PENT	IVIg, steroid, PE	d12	7 mg/kg/day	270 days	yes
27		5	F	PENT, PRP, KE, CBD	IVIg, steroid, PE, rituximab	d34	9 mg/kg/day	200 days	yes
28		14	F	PENT	IVIg, steroid, KD	d12	7.5 mg/kg/day	183 days	yes
29		5	F	PENT, clonidine	KD	d1	4.7 mg/kg/day	14 days	-
30		16	F	MDZ, KETA, CBD	IVIg, steroid, PE, rituximab, KD	d30	2 mg/kg/day	97 days	yes
31		5	M	MDZ, PENT, KE	IVIg, steroid, PE, KD	d14	5 mg/kg/day	2 days	-
32		4	M	MDZ, PENT, KE, CBD	IVIg, steroid, PE, rituximab	d33	5 mg/kg/day	5 days	no

Supplemental Table 1 Clinical features and Treatment of Febrile Infection-Related Epilepsy Syndrome

Abbreviations: F, female; M, male; ND, not described ASM, anti-seizure medication; CBD, cannabinoids; cEEG, continuous electroencephalography; DBS, deep brain stimulation; DRESS, drug reaction with eosinophilia and systemic symptoms syndrome; hypothermia, HYPO; Ketamine, KE
IVIG, intravenous immunoglobulin; isoflurane, ISO; KD, ketogenic diet; LFT, liver function test; MDZ, midazolam; Pentobarbital, PENT, Thiopentone, plasmapheresis,PE TH; Sz, seizure Propofol,PRP Oxcarbazepine, OXC
ND# The individual prognosis is not known but is being considered as a cohort study.
Special additions* Immune therapy including IVIg, steroid, or CMN-DBS, KD, HYPO, PE
